# C1q/TNF-Related Protein 9 Protects Diabetic Rat Heart against Ischemia Reperfusion Injury: Role of Endoplasmic Reticulum Stress

**DOI:** 10.1155/2016/1902025

**Published:** 2016-10-04

**Authors:** Sanxing Bai, Liang Cheng, Yang Yang, Chongxi Fan, Dajun Zhao, Zhigang Qin, Xiao Feng, Lin Zhao, Jipeng Ma, Xiaowu Wang, Jian Yang, Xuezeng Xu, Dinghua Yi, Wei Yi

**Affiliations:** ^1^Department of Cardiovascular Surgery, Xijing Hospital, The Fourth Military Medical University, 127 Changle West Road, Xi'an 710032, China; ^2^Department of Biomedical Engineering, The Fourth Military Medical University, 169 Changle West Road, Xi'an 710032, China; ^3^Department of Thoracic Surgery, Tangdu Hospital, The Fourth Military Medical University, 1 Xinsi Road, Xi'an 710038, China

## Abstract

As a newly identified adiponectin paralog, C1q/TNF-related protein 9 (CTRP9) reduces myocardial ischemia reperfusion (IR) injury through partially understood mechanisms. In the present study, we sought to identify the role of endoplasmic reticulum stress (ERS) in CTRP9 induced cardioprotection in diabetic heart. Isolated hearts from high-fat-diet (HFD) induced type 2 diabetic Sprague-Dawley rats were subjected to ex vivo IR protocol via a Langendorff apparatus at the presence of globular CTRP9. CTRP9 significantly improved post-IR heart function and reduced cardiac infarction, cardiomyocytes apoptosis, Caspase-3, Caspase-9, Caspase-12, TNF-*α* expression, and lactate dehydrogenase activity. The cardioprotective effect of CTRP9 was associated with reduced ERS and increased expression of disulfide-bond A oxidoreductase-like protein (DsbA-L) in diabetic heart. CTRP9 reduced ERS in thapsigargin (TG) treated cardiomyocytes and protected endoplasmic reticulum (ER) stressed H9c2 cells against simulated ischemia reperfusion (SIR) injury, concurrent with increased expression of DsbA-L. Knockdown of DsbA-L increased ERS and attenuated CTRP9 induced protection against SIR injury in H9c2 cells. Our findings demonstrated for the first time that CTRP9 exerts cardioprotection by reducing ERS in diabetic heart through increasing DsbA-L.

## 1. Introduction

Patients with type 2 diabetes have increased risk of developing ischemic heart disease and more severe and fatal myocardial infarctions than nondiabetic population [1, 2]. An understanding of the internal link between type 2 diabetes and cardiovascular injury may help identifying novel therapies alleviating ischemic myocardial injury to reduce cardiovascular morbidity and mortality.

Adiponectin (APN) is an adipocytokine with a collagenous domain and a C-terminal globular domain and was predominantly secreted by adipose tissue [3]. Studies have revealed that APN protects against myocardial IR injury, while the cardioprotective effect is attenuated in diabetic condition [4–6]. In search for molecules with structure similarity to APN, C1q/TNF-related proteins (CTRPs) are identified. This newly discovered APN paralog family consists of at least fifteen (CTRP1-CTRP15) family members and has been demonstrated to have diverse functions similar to APN [7–9]. Among all the CTRP family members, CTRP9 shows the highest amino acid identity to APN, which consists of 4 distinct domains, including an N-terminal signal peptide, a short variable domain, a collagen-like domain, and a C-terminal C1q-like globular domain. CTRP9 is mainly expressed in fat tissue, forms heterotrimer with APN, and acts as an adipocytokine that exerts a beneficial effect on glucose metabolism [10]. CTRP9 is reported to protect against IR injury [11, 12] and to reduce postmyocardial infarction heart remolding [13]. However, unlike APN, CTRP9 protects diabetic heart against IR injury [14], but with partially understood mechanisms.

ERS played important role in the pathogenesis of type 2 diabetes [15, 16], and suppressed ERS contributed to reduced cardiac infarct size in HFD induced type 2 diabetes [17]. CTRP9 was reported to inhibit ERS in hepG2 cells [18]; however, whether ERS was involved in CTRP9 induced cardioprotection in diabetic heart has never been investigated.

The aim of the present study was to investigate the role of ERS in CTRP9 induced cardioprotection against IR injury in HFD induced type 2 diabetic heart and the underlying mechanism.

## 2. Materials and Methods

This study was performed according to the Guide for the Care and Use of Laboratory Animals that was published by the US National Institutes of Health (National Institutes of Health Publication number 85-23, revised 1996) and was approved by the Ethics Committee of the Fourth Military Medical University.

### 2.1. Experimental Animal

All of the experiments were performed on healthy adult male Sprague-Dawley (SD) rats that weighed between 100 and 120 g and were obtained from the animal center of the Fourth Military Medical University. All the rats were kept in a pathogen-free environment with free access to food and tap water. Diabetic rats were provided with HFD (standard diet supplemented with 10% sugar, 10% lard, 2% cholesterol, and 0.5% bile acid, with 60% kilocalories from fat) for 8 weeks and were given a single shot of streptozotocin (STZ, Sigma-Aldrich, 30 mg/Kg) intraperitoneally one week before the experiment [19, 20]. Control rats were provided with standard diet and intraperitoneally injected with equal amount of saline.

### 2.2. Materials

Dulbecco's modified Earle's medium (DMEM) and fetal bovine serum (FBS) were purchased from Thermo Fisher Scientific (Grand Island, NY, USA). Streptozotocin (STZ) and thapsigargin (TG) were purchased from Sigma-Aldrich (St. Louis, MO, USA). Antibodies against disulfide-bond A oxidoreductase-like protein (DsbA-L) and CTRP9 were bought from Santa Cruz Biotechnology (Santa Cruz, CA, USA); antibodies against CHOP, GRP-78, TNF-*α*, Caspase-3, Caspase-9, Caspase-12, and GAPDH were from Cell Signaling Technology (Beverly, MA, USA). The terminal deoxynucleotidyl transferase dUTP nick-end labeling (TUNEL) kits were purchased from Roche Diagnostics (Mannheim, Germany). Globular CTRP9 proteins were purchased from Aviscera Bioscience (Santa Clara, CA, USA). Lactate dehydrogenase (LDH) assay kit was purchased from the Institute of Jiancheng Bioengineering (Nanjing, Jiangsu, China). The rat insulin ELISA kit was brought from Shanghai Enzyme-linked Biotechnology (Shanghai, China). The BCA kit was purchased from Thermo Fisher (Waltham, MA, USA). The rabbit anti-goat, goat anti-rabbit, and goat anti-mouse secondary antibodies were purchased from Zhongshan Company (Beijing, China).

### 2.3. Langendorff Perfused Rat Heart

The Langendorff perfused rat heart model was established as previously described [21–23]. Briefly, the rat was sacrificed after anesthesia and the heart was mounted on a glass needle and perfused retrogradely with a noncirculating Langendorff apparatus (Radnoti Glass Technology Inc., USA) through the ascending aorta with KH buffer at constant pressure (i.e., 90 cm H_2_O). The perfusate was maintained at 37°C by a circulating water bath and gassed continuously with 5% CO_2_ and 95% O_2_. The KH buffer solution used for heart preparation and perfusion contained the following reagents (final concentrations in mM): NaCl 118, NaHCO_3_ 25, MgSO_4_ 1.2, CaCl_2_ 1.25, KCl 4.7, KH_2_PO_4_ 1.2, and glucose 11, with pH adjusted to 7.4 with 1 M HCl. A water-filled latex balloon coupled to a pressure transducer (Model 100 BP-Biopac System Inc., USA) was inserted into the left ventricular cavity via the left auricle to record pressure. At the beginning of the experiment, the left ventricular end-diastolic pressure (LVEDP) was adjusted to approximately 5 mmHg by inflating the balloon. The hemodynamic parameters, including heart rate (HR), left ventricular developing pressure (LVDP), and maximum and minimum electronically differentiated derivative of left ventricular pressure (±d_*P*_/d_*t*_), were recorded and stored with the AcqKnowledge 3.8.1 software package and a Biopac Data Acquisition System (Biopac Systems Inc., USA). The rate-pressure product (RPP = LVDP × HR) was calculated as an index of contractility [24–26]. Langendorff perfused hearts were allowed to equilibrate until heart rate and contractility reached steady state (i.e., 30 minutes or more) and were subjected to different experimental protocols as described. Control hearts were perfused with Krebs-Henseleit (KH) buffer continuously for 120 minutes. IR protocol consists of 30 minutes of global ischemia followed by 60 minutes of reperfusion after equilibration. Globular CTRP9 dissolved in 1 mL of KH buffer or equal amount of KH buffer was acutely administered as 1 mL bolus injections into the perfusate via an injection port positioned upstream of the heart at the end of equilibration. At the end of reperfusion, the hearts were removed from the Langendorff apparatus and weighted for cardiac mass index (wet heart weight/tibia length) and were stored at −80°C immediately for further analysis.

### 2.4. Metabolic Characterization

All the rats were fasted overnight by removal to a clean cage without food at the end of 8 weeks and were weighed the next morning; 30 *μ*L blood was obtained via tail clip to assess fasting plasma glucose with a glucose meters and fasting plasma insulin with the rat insulin ELISA kit. The Homeostatic Model Assessment (HOMA) score, a surrogate measure of insulin resistance, was calculated via HOMA calculator version 2.2.2 (University of Oxford, UK) [13]. Only those animals showing hyperglycemia (fasting blood glucose level ≥11.1 mM/L for at least three samples) at week 8 were considered to have developed type 2 diabetes.

### 2.5. Measurement of LDH Release

The levels of LDH in the coronary effluent were determined using a LDH colorimetric assay kit according to the manufacturer's instructions. The LDH release that accumulated during the 60 minutes of reperfusion was obtained by calculating the total amount of LDH from the coronary effluent of individual 30-second collections. The activity of LDH was normalized against the coronary flow and was expressed as milliunit/mL [27].

### 2.6. Quantization of Apoptotic Cardiomyocytes

Myocardium slices or cardiomyocytes slides were evaluated immunohistochemically to determine the percentage of cells exhibiting apoptotic-positive staining. A double-staining technique was used: TUNEL staining was used to quantitate apoptotic cell nuclei and 6-diamidino-2-phenylindole (DAPI) staining was used to quantitate the total myocardial cell nuclei. The TUNEL-positive cells that showed green nuclear staining and all of the cells with blue nuclear DAPI staining were counted within five randomly chosen fields under a high power magnification. The percentage of apoptotic cells was expressed as the ratio of positively stained apoptotic myocytes/the total number of myocytes counted ×100% [6, 28–30].

### 2.7. Quantization of Myocardial Infarct Size

The myocardial infarct size was detected using TTC method as described previously [29–31]. Briefly, frozen ventricles were sliced into uniform sections of about 1-2 mm thickness. The slices were incubated in 1% w/v triphenyltetrazolium chloride (TTC) stain at 37°C in 0.2 M Tris-chloride buffer for 30 minutes to demarcate the viable and nonviable myocardium. The normal myocardium was stained brick red while the infarcted portion remained unstained. A blinded technician assessed the percentage of infarcted area using computer-assisted planimetry (OPTIMAS version 5.2). In this study, the hearts were subjected to global ischemia; therefore, the entire ventricle was considered to be the area at risk. The normalized infarct size was expressed as the percentage of infarct size/area at risk.

### 2.8. Cell Culture and Stimulated Ischemia Reperfusion

The H9c2 rat cardiomyocyte cell line was purchased from the American Type Culture Collection. Cells were cultured with DMEM containing 4.5 g/L D-glucose, 3.7 g/L sodium bicarbonate, and 110 mg/L sodium pyruvate, supplemented with 10% fetal bovine serum in a humidified incubator with 95% air and 5% CO_2_ at 37°C. The culture medium was changed every two or three days. Cells were split when a confluency of ~70% was achieved using trypsin EDTA (Lonza) and subcultured at a ratio of 1 : 3. H9c2 cells were subjected to simulated ischemia reperfusion (SIR) as described in our previously published study [19]. In brief, cells were incubated with a simulated ischemic buffer [137 NaCl, 12 KCl, 0.49 MgCl_2_, 0.9 CaCl_2_, 4 HEPES, 10 deoxyglucose, 0.75 sodium dithionate, and 20 lactate (in mM/L)] for 2 h in a humidified cell culture incubator (95% air and 5% CO_2_ at 37°C). After that, reperfusion was performed by returning the cells to normal culture medium for 4 h in a humidified cell culture incubator (95% air and 5% CO_2_ at 37°C). In some of the experiments, H9c2 cells were pretreated with globular CTRP9 (1 ug/mL) or equal amount of saline for 24 h followed with TG (0.01 *μ*M/L) cotreatment for 24 h.

### 2.9. RNA Interference and Generation of DsbA-L-Suppressed Cells

The sense and antisense sequences of siRNA were chemically synthesized and ligated into the pSIREN-RetroQ-ZsGreen Vector (*BamH* I/*EcoR* I, TaKaRaBiotechnology, Dalian, China). The sequences for siRNA and scrambled control are 5′-GGTCCTATGCAGATACCAA-3′ and 5′-AGTTCAACGACCAGTAGTC-3′, respectively. These recombinant plasmids vectors independently express a green fluorescent protein (GFP), and, as a result, transfected cells emit green fluorescence. For H9c2 cardiomyocytes transfection, the plasmids were acquired in supercoil form using a large scale plasmid extraction kit (EndoFree Plasmid kit, Tiangen, Beijing, China) and confirmed by sequencing. A day before transfection, H9c2 cells were cultured in 6-well culture plates in culture medium without antibiotics at a density of 5 × 10^5^/well. On reaching 80–90% confluence, transfection of aforementioned vectors was performed using Lipofectamine 3000 Transfection Reagent (Invitrogen) according to the manufacturer's instructions. The expression of GFP was observed under fluorescent microscopy 48 h after transfection [32, 33].

### 2.10. Western Blot

The protein samples were prepared as previously described. Briefly, the cardiac tissues were immediately frozen at −70°C after reperfusion and were homogenized in lysis buffer containing 50 mM/L Tris–HCl (pH7.3), 150 mM/L NaCl, 5 mM/L EDTA, 1 mM/L dithiothreitol, 1% Triton X-100, and 1% protease inhibitor cocktail. Cells were washed with cold PBS twice after treatment and then harvested in lysis buffer containing a cocktail of protease and phosphatase inhibitors, dithiothreitol, trichostatin-A, and Triton X-100 (0.1%), using 100 *μ*L of the lysis buffer per 35 mm dish. The lysates were centrifuged for 15 minutes at 12,000 ×g, and the resulting supernatant was transferred to a new tube and stored at −70°C. The protein concentrations were determined using a BCA kit, and the proteins were separated by electrophoresis and transferred to PVDF membranes. The membranes were blocked for 1 h in Tris-buffered saline and Tween 20 (TBST, pH 7.6) that contained 5% nonfat dry milk and then incubated overnight at 4°C with antibodies against GAPDH (1 : 5000), CHOP (1 : 2000), GRP78 (1 : 1000), DsbA-L (1 : 2000), Caspase-3 (1 : 1000), Caspase-9 (1 : 1000), Caspase-12 (1 : 1000), TNF-*α* (1 : 1000), and CTRP9 (1 : 1000) followed by washes with TBST. The membranes were then probed with appropriate secondary antibodies (1 : 5000) at room temperature for 90 min, followed by washes with TBST. The protein bands were detected by chemiluminescence and were quantified using the Quantity One software package (Bio-Rad Laboratories, UK).

### 2.11. Statistical Analysis

All of the values are presented as the mean ± the standard error of the mean (SEM). Group comparisons were performed using an ANOVA (GraphPad Prism 5). All of the groups were analyzed simultaneously with a LSD *t*-test. A difference of *p* < 0.05 was considered to be statistically significant.

## 3. Results

### 3.1. CTRP9 Protected Diabetic Hearts against IR Injury

After 8 weeks of feeding with HFD and a single STZ injection, typical type 2 diabetes was developed. As shown in Table 1, both normal and type 2 diabetic rats had similar body weight and cardiac mass index; however, diabetic rats had significantly elevated fasting blood glucose and HOMA value compared with normal rats. Both normal and diabetic rats had similar fasting blood insulin level. These data suggested HFD induced type 2 diabetes was developed.

**Figure 1:**
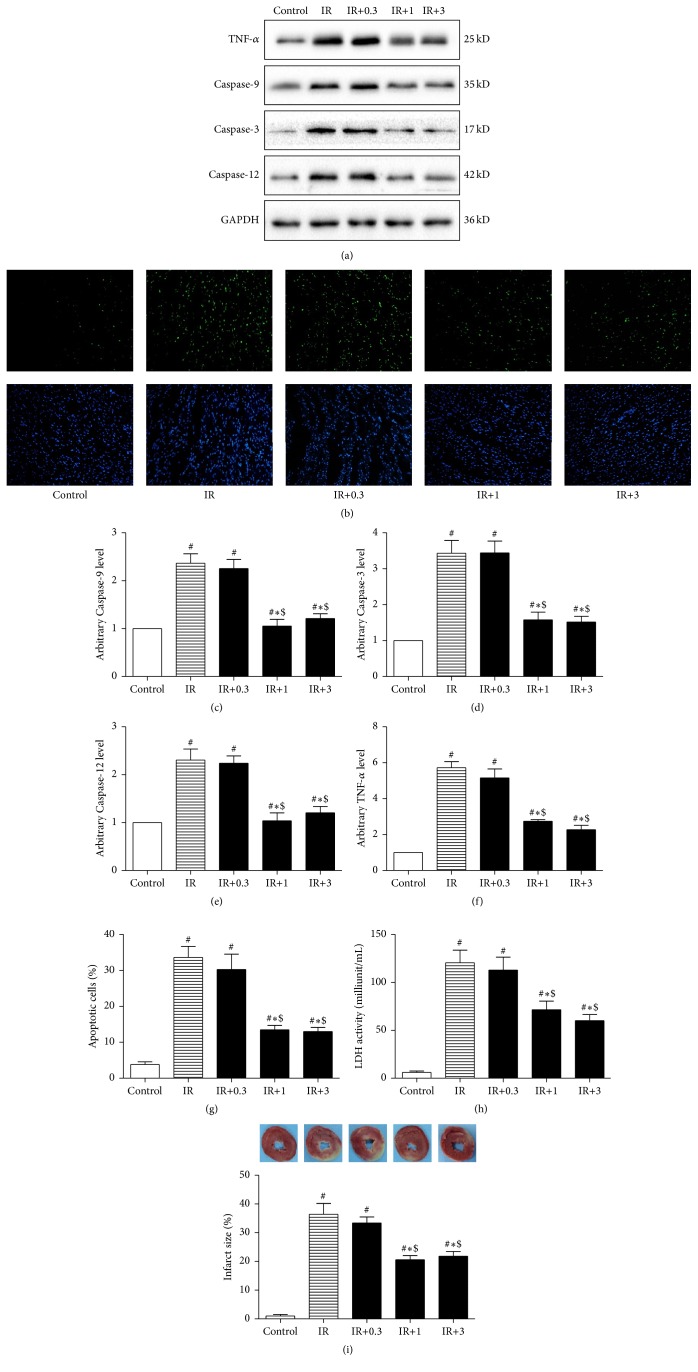
CTRP9 protected diabetic heart against IR injury. Isolated hearts from HFD induced type 2 diabetic rats were subjected to control or IR protocol at the presence of different dosage of globular CTRP9 or equal amount of KH buffer. Cardiomyocytes apoptosis was determined by TUNEL staining and Caspase-3, Caspase-9, Caspase-12, and TNF-*α* ((c), (d), (e), and (f)) were examined by Western blot. GAPDH was used as loading control. Representative images (b) and bar graph (g) of apoptotic cardiomyocytes were shown. The apoptotic cells were detected by immunofluorescent staining with TUNEL ((b), upper panel), and DAPI staining was used to label the nuclei ((b), lower panel). Infarct size was determined by TTC staining ((i), upper panel) and was expressed as the percentage of infarct size/area at risk ((i), lower panel). LDH activity in the coronary effluent was measured and was expressed as milliunit/mL (h). The results were expressed as the mean ± SEM, *n* = 8 per group. ^#^ *p* < 0.01 compared with control; ^*∗*^ *p* < 0.01 compared with IR, ^$^ *p* < 0.01 compared with IR+0.3.

We next investigate the protective effect of different dosage of globular CTRP9 against IR injury in diabetic heart. Isolated hearts from type 2 diabetic rats were subjected to ex vivo IR or control perfusion at the absence or presence of different dosage of CTRP9. All the isolated hearts had similar basal conditions at the end of equilibration (30 min) as summarized in Table 2. IR triggered inflammatory response in isolated heart as shown by significantly increased TNF-*α* expression (Figures 1(a) and 1(e)). IR also increased cardiomyocytes apoptosis as evidenced by significantly increased percentage of apoptotic cells and Caspase-3, Caspase-9, and Caspase-12 expression (Figures 1(a)–1(f)). IR significantly deteriorated cardiac function as demonstrated by significantly decreased CF, LVDP, ±d_*P*_/d_*t*_, and RPP at the end of reperfusion (Table 2). IR induced dramatic cardiac injury as shown by significantly increased infarct size and LDH activity compared with that of control (Figures 1(h) and 1(i)).

At the dosage of 0.3 *μ*g/mL, CTRP9 had no measurable protective effect compared with KH buffer. Specifically, there was no significant change of LVDP, ±d_*P*_/d_*t*_, RPP, infarct size, LDH release, cardiomyocytes apoptosis, Caspase-3, Caspase-9, Caspase-12, and TNF-*α* expression, except that CF was significantly increased (Table 2). Noticeably, at the dosage of 1 *μ*g/mL, CTRP9 significantly improved the post-IR CF, LVDP, ±d_*P*_/d_*t*_, and RPP (Table 2) and reduced infarct size, LDH level, cardiomyocytes apoptosis, Caspase-3, Caspase-9, Caspase-12, and TNF-*α* level (Figure 1) compared with control. However, further increase of CTRP9 to 3 *μ*g/mL failed to induce further improvement of CF, LVDP, ±d_*P*_/d_*t*_, and RPP, or further reduction of infarct size, LDH level, apoptotic cell rate, Caspase-3, Caspase-9, Caspase-12, and TNF-*α* expression. These data suggested that CTRP9 at the dosage of 1 *μ*g/mL was enough to protect diabetic hearts against IR induced injury. And the dosage of 1 *μ*g/mL was used in the following experiment.

### 3.2. CTRP9 Reduced ERS in Diabetic Hearts

**Figure 2:**
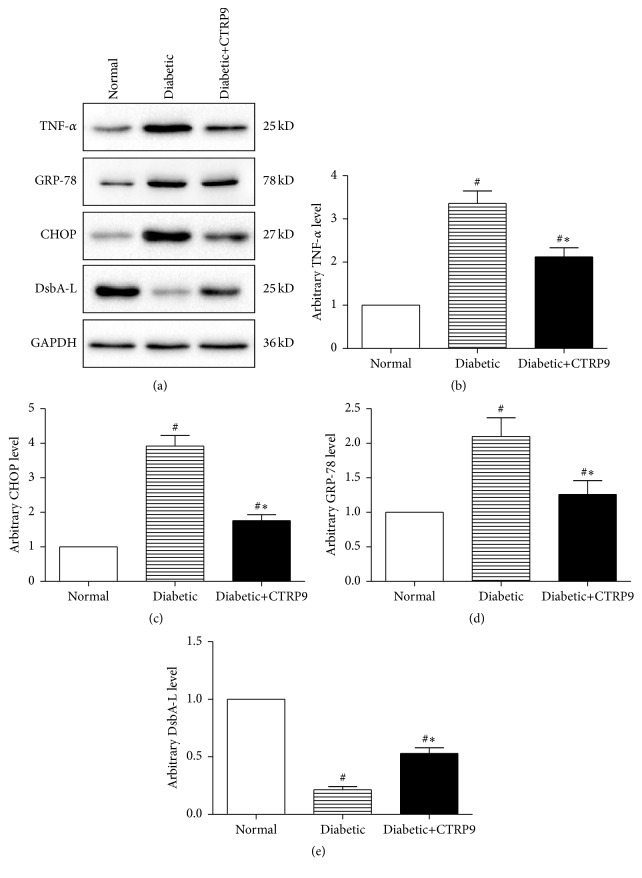
CTRP9 reduced ERS in diabetic heart. Isolated hearts from normal or HFD induced type 2 diabetic rats were subjected to IR protocol with globular CTRP9 (1 *μ*g/mL) or equal amount of KH buffer. Representative images (a) and bar graphs of cardiac TNF-*α* (b), CHOP (c), GRP-78 (d), and DsbA-L (e) determined by Western blots. GAPDH was used as loading control. The results were expressed as the mean ± SEM, *n* = 8 per group. ^#^ *p* < 0.01 compared with normal heart; ^*∗*^ *p* < 0.01 compared with diabetic heart treated with KH buffer.

We next examined the effect of CTRP9 on ERS. Both normal and diabetic hearts were subjected to ex vivo IR with or without CTRP9. As depicted in Figure 2, western blot analysis of isolated perfused heart homogenates revealed that ERS was significantly increased in diabetic hearts after IR, as demonstrated by significantly increased ERS markers, glucose-regulated protein, 78 kD (GRP-78), and C/EBP-homologous protein (CHOP) expression compared with normal hearts (Figures 2(a), 2(c), and 2(d)). And DsbA-L (previously named as GST Kappa), a newly identified endoplasmic reticulum associated protein chaperone interacting with adiponectin, was significantly reduced in diabetic heart (Figures 2(a) and 2(e)). Inflammatory response was also activated in diabetic heart after IR as shown by significantly increased expression of TNF-*α* (Figures 2(a) and 2(b)). CTRP9 significantly suppressed ERS in diabetic hearts as demonstrated by significantly reduced GRP-78 and CHOP expression compared with treating with KH buffer, together with significantly increased DsbA-L expression (Figures 2(a), 2(c), 2(d), and 2(e)). CTRP9 also reduced the expression of inflammatory factor TNF-*α* (Figures 2(a) and 2(b)). These data suggested that CTRP9 decreases ERS, alleviates inflammatory response, and increases DsbA-L expression in isolated perfused diabetic hearts.

### 3.3. CTRP9 Protected ER Stressed H9c2 Cardiomyocyte against SIR Injury

**Figure 3:**
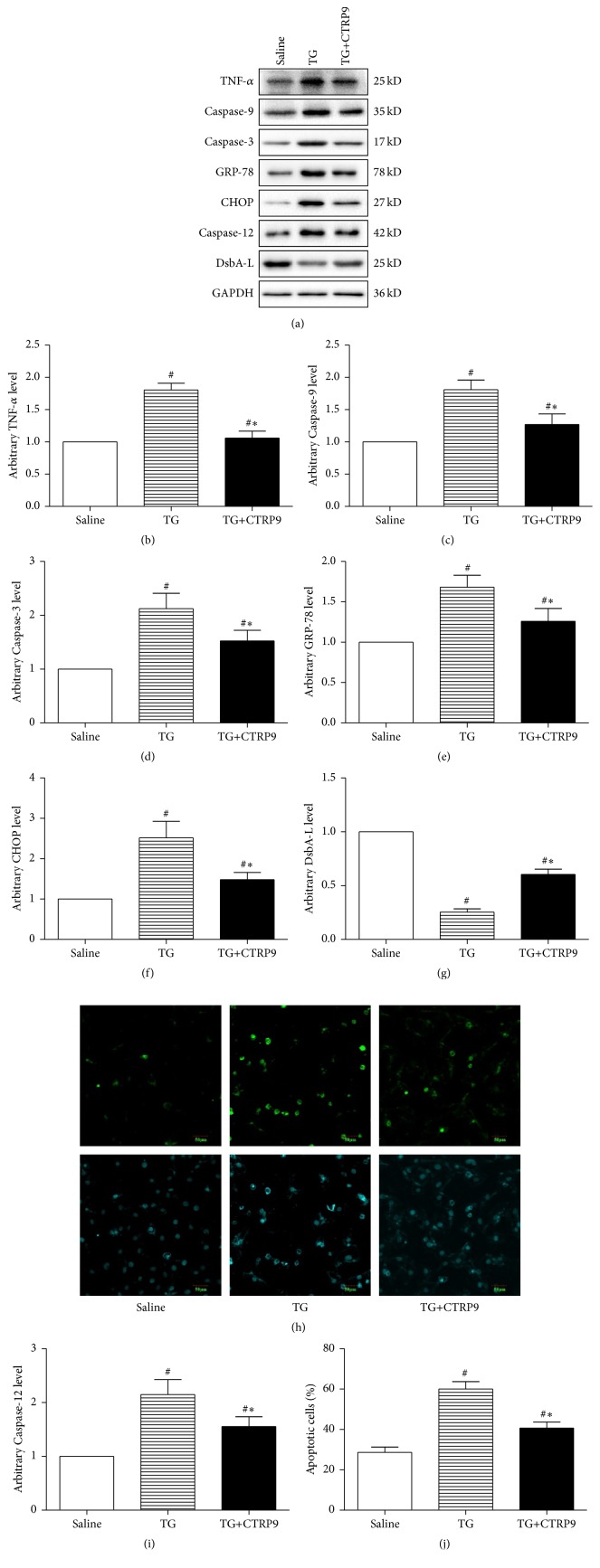
CTRP9 protected ER stressed H9c2 cardiomyocytes against SIR injury. H9c2 cells were pretreated with globular CTRP9 (1 *μ*g/mL) or equal amount of saline for 24 h followed with TG (0.01 *μ*M/L) cotreatment for 24 h. Representative images (a) and bar graphs of TNF-*α* (b), Caspase-9 (c), Caspase-3 (d), Caspase-12 (i), GRP-78 (e), CHOP (f), and DsbA-L (g) and in H9c2 cardiomyocyte determined by Western blots. GAPDH was used as loading control. Representative images (h) and bar graph (j) of apoptotic cardiomyocytes were shown. The apoptotic cells were detected by immunofluorescent staining with TUNEL ((h), upper panel), and DAPI staining was used to label the nuclei ((h), lower panel). The results were expressed as the mean ± SEM, *n* = 6 per group. ^#^ *p* < 0.01 compared with cells treated with saline; ^*∗*^ *p* < 0.01 compared with cells treated with TG.

In order to confirm the role of ERS in CTRP9 induced cardiac protection, we next investigate the protective effect of globular CTRP9 against SIR injury in ER stressed cardiomyocytes. H9c2 cells treated with saline or TG, a chemical widely used to stimulate ERS by inhibiting ER calcium-ATPase [34], were subjected to SIR at the presence or absence of globular CTRP9 (1 *μ*g/mL) [18]. TG treatment led to significantly increased ERS and inflammatory response, as demonstrated by enhanced CHOP, GRP-78, and TNF-*α* expression, together with greatly reduced cellular protein levels of DsbA-L (Figures 3(a), 3(b), 3(e), 3(f), and 3(g)). TG treated H9c2 cells suffered severer inflammatory response and cell injury as evidenced by significantly increased expression of TNF-*α*, Caspase-3, Caspase-9, and Caspase-12 and higher percentage of apoptotic cells (Figures 3(a)–3(d) and 3(h)–3(j)) compared with cells treated with saline. CTRP9 partially reduced TG induced ERS and inflammatory response in H9c2 cell as demonstrated by significantly reduced CHOP, GRP-78, and TNF-*α* expression, concurrent with significantly increased DsbA-L expression compared with treating with vehicle. More importantly, CTRP9 protected TG treated cardiomyocytes against SIR injury as shown by significantly reduced percentage of apoptotic cells and Caspase-3, Caspase-9, and Caspase-12 expression compared with treating with vehicle. These data suggested that ERS was involved in CTRP9 induced protection against SIR injury in cardiomyocytes.

### 3.4. DsbA-L Knockdown Attenuated CTRP9 Induced Protection

**Figure 4:**
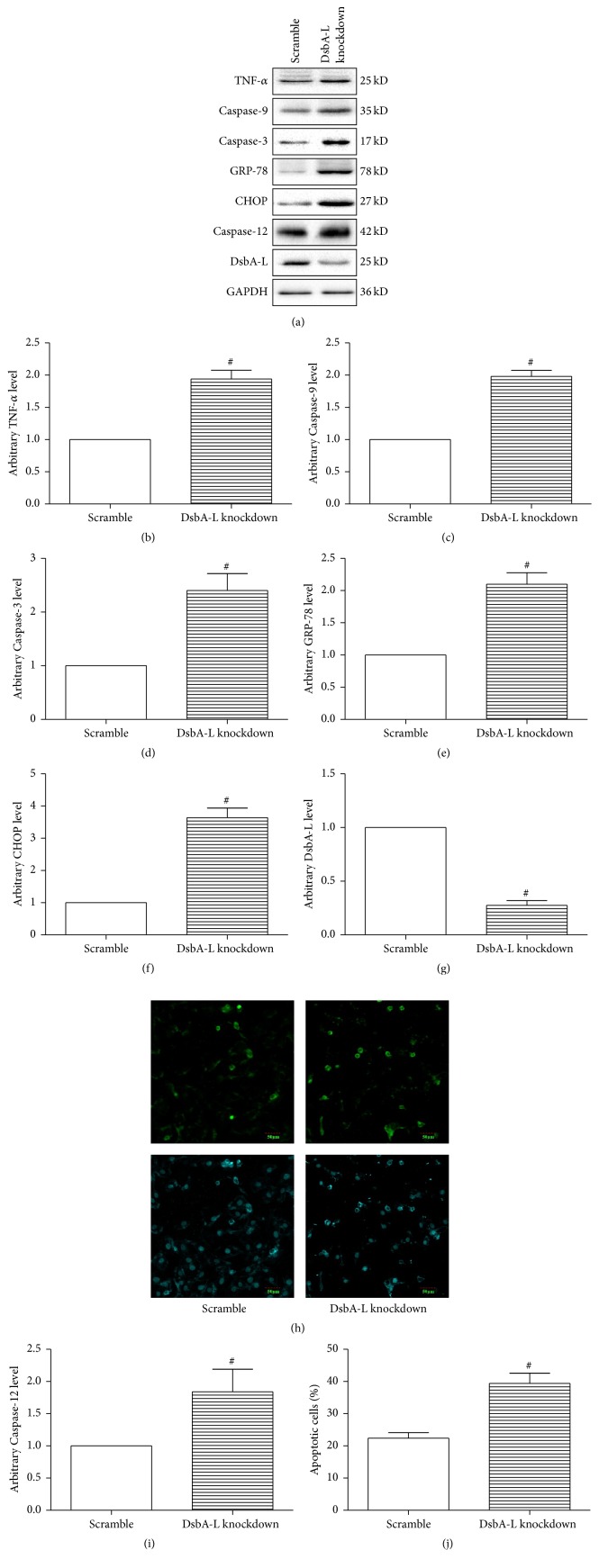
DsbA-L knockdown increased ERS in H9c2 cells and attenuated CTRP9 induced protection against SIR injury. Both scramble and DsbA-L suppressed cells were subjected to SIR injury at the presence of globular CTRP9 (1 *μ*g/mL). Representative images (a) and bar graphs of TNF-*α* (b), Caspase-9 (c), Caspase-3 (d), Caspase-12 (i), GRP-78 (e), CHOP (f), and DsbA-L (g) in H9c2 cardiomyocytes were determined by Western blots. GAPDH was used as loading control. Representative images (h) and bar graph (j) of apoptotic cardiomyocytes were shown. The apoptotic cells were detected by immunofluorescent staining with TUNEL ((h), upper panel), and DAPI staining was used to label the nuclei ((h), lower panel). The results were expressed as the mean ± SEM, *n* = 6 per group. ^#^ *p* < 0.01 compared with scramble.

DsbA-L, a newly identified ER chaperone that facilitates correct folding and assembly of proteins in the ER, was expressed in a number of tissues and its expression level is closely related to obesity/diabetes [35]. Downregulation of DsbA-L led to accumulation of incorrectly folded proteins that in turn induces UPR [36], and ERS was reported to increase in DsbA-L suppressed 3T3 cells. Together with our previous results that DsbA-L was significantly reduced in diabetic hearts and ER stressed cardiomyocytes, which could be partially reversed by CTRP9, we hypothesized that DsbA-L may play a role in CTRP9 induced cardioprotection. We next subjected scramble or DsbA-L suppressed H9c2 cells to SIR at the presence of globular CTRP9. The expression of GFP was observed under fluorescent microscopy beginning 48 h after transfection (Supplementary Figure  1, in Supplementary Material available online at http://dx.doi.org/10.1155/2016/1902025), and then the protein levels of DsbA-L were detected by Western blot (Figures 4(a) and 4(g)). Since DsbA-L protein expression level was reduced to approximately 30% of normal in diabetic heart, the concentration of plasmid employed was adjusted to reduce H9c2 cell DsbA-L expression to a level comparable to that observed in the diabetic heart. DsbA-L knockdown induced ERS in H9c2 cells as shown by significantly increased CHOP and GRP-78 expression (Figures 4(a), 4(e), and 4(f)). TNF-*α* expression was also increased in DsbA-L suppressed cells (Figures 4(a) and 4(b)). More importantly, suppression of DsbA-L dramatically attenuated CTRP9 induced cardioprotection against SIR injury in H9c2 cells as demonstrated by significantly increased percentage of apoptotic cells and Caspase-3, Caspase-9, and Caspase-12 expression compared with control (Figures 4(a), 4(c), 4(d), 4(h), 4(i), and 4(j)). These data suggested that DsbA-L played a role in CTRP9 induced suppression of ERS.

## 4. Discussion

CTRPs are newly discovered adipocytokines that closely related to diabetes and cardiovascular diseases. We found in this study that CTRP9 level was reduced in diabetic heart, in ER stressed and DsbA-L suppressed cardiomyocytes (Supplementary Figure  2), replenishing of CTRP9 protected diabetic heart, and ER stressed cardiomyocytes against IR injury. DsbA-L suppression attenuated CTRP9 cardioprotection.

In order to find out an appropriate dosage, different dosages of CTRP9 were administrated to isolated perfused rat heart. At the dosage of 0.3 *μ*g/mL, CTRP9 had no detectable protective effect, except that the post-IR coronary flow was significantly improved. This may be a secondary effect of dilated coronary artery, as CTRP9 was reported to induce vasodilation with potency exceeding that of APN through AMPK/eNOS/NO [37]. Measurable cardiac protection was observed when CTRP9 was increased to 1 *μ*g/mL. However, further increase of dosage failed to induce further improvement of heart function or reduction of cardiac injury. Our results were in agreement with Su et al. [14] that CTRP9 protected diabetic heart against IR injury.

ER is a membrane-bound and structurally intricate organelle present in all eukaryotic cells and is the major place for the synthesis of proteins and lipids and internal calcium storage [38, 39]. Obesity and type 2 diabetes induced low-grade chronic inflammation and hypoxic microenvironment can lead to abnormal accumulation of misfolded proteins in the ER lumen [40, 41]. The folding capacity of the ER fails to accommodate the load of unfolded proteins; ER homeostasis is perturbed to a condition referred to as ERS. In response to ERS, an adaptive mechanism termed the unfolded protein response (UPR) is implemented to reestablish homeostasis in the ER [40–43]. Several studies reported that AMPK was involved in reducing ERS [17, 44]. Although this study focused on the role of ERS in CTRP9 cardioprotection, further study needs to be done to investigate the role of AMPK and the relationship between AMPK and ERS in CTRP9 induced cardioprotection.

We found in the present study that ERS and inflammatory response were markedly increased in diabetic heart after IR, which was in agreement with previous observations that ERS and inflammation were involved in diabetic cardiac dysfunction [15]. More importantly, we found that CTRP9 significantly reduced ERS and inflammatory factor expression in diabetic heart, concurrent with increased prosurvival molecular DsbA-L. Together with previous findings that CTRP9 inhibits ERS in hepatic cells [18], these data favor the notion that reducing ERS and inflammation protect against IR induced injury in diabetic animal [15–17]. The observation we made that CTRP9 protected ER stressed H9c2 cells against SIR injury further confirmed this notion.

DsbA-L is a recently identified ER associated protein chaperone that colocalized with the ER marker protein disulfide isomerase and the mitochondrial marker [35, 45]. DsbA-L is expressed in various tissues such as adipose, liver, kidney, pancreas, and the heart. The cellular level of DsbA-L is negatively correlated with obesity in mice and human, and its expression is stimulated by insulin sensitizer rosiglitazone but inhibited by the inflammatory cytokine TNF-*α* [35]. Our study found that DsbA-L was markedly decreased in diabetic heart and suppression of DsbA-L induced ERS and inflammatory response in H9c2 cardiomyocytes. However, CTRP9 was capable of increasing the expression of DsbA-L in both diabetic heart and TG treated H9c2 cardiomyocytes, concurrent with reduced ERS and inflammatory response. Together with previous observation that reduced DsbA-L activates ERS in 3T3-L1 adipocytes [36], it is highly likely that DsbA-L functions as a chaperone to facilitate correct folding of macromolecules in the ER and plays key role in maintaining cell homeostasis. Thus, reduced expression of DsbA-L may lead to overloading of unfolded proteins, resulting in increased ERS.

In conclusion, the present study found that administration of CTRP9 at the onset of ischemia protected diabetic heart against IR injury. CTRP9 induced cardioprotection was associated with reduced ERS and inflammatory response in DsbA-L-dependent mechanism.

## Supplementary Material

Forty-eight hours after DsbA-L RNAi recombinant plasmids and non-specific scramble sequence were transfected in rat cardiomyocytes, transfection efficiency was shown as the expression of GFP in the recombinant plasmids (Supplementary Figure 1). Isolated hearts from normal or HFD induced type 2 diabetic rats were subjected to IR protocol and cardiac CTRP9 expression were examined at the end of reperfusion (Supplementary Figure 2A). TG or saline treated cardiomyocytes were subjected to SIR and cellular CTRP9 expression were examined at the end of reperfusion (Supplementary Figure 2B). Cardiomyocytes transfected with DsbA-L RNAi recombinant plasmids or non-specific scramble sequence were subjected to SIR and cellular CTRP9 expression were examined at the end of reperfusion (Supplementary Figure 2C). Representative images (upper panel) and bar graphs (lower panel) of cardiac CTRP9 determined by Western blots were shown. GAPDH was used as loading control. The results were expressed as the mean ± SEM, *n* = 8 per group, ^#^ *p* < 0.05.

## Figures and Tables

**Table 1:** Development of type 2 diabetes in male SD rats.

Parameters	Normal	Diabetic
Body weight (g)	326 ± 12.5	325 ± 5.6
Cardiac mass index (g/cm)	320 ± 9.0	307 ± 4.9
Fasting blood glucose (mmol/L)	8.0 ± 0.20	21.6 ± 0.71^*∗*^
Fasting plasma insulin (pmol/L)	175.7 ± 3.16	178.1 ± 2.90
HOMA score	4.2 ± 0.09	20.3 ± 3.31^*∗*^

Diabetic rats were provided with HFD and were given a single shot STZ (30 mg/Kg) intraperitoneally. Control rats were provided with standard diet and injected with equal amount of saline. All the rats were fasted overnight and body weight, cardiac mass index, fasting blood glucose, fasting plasma insulin, and the HOMA score were determined. Cardiac mass index = wet heart weight/tibia length, ^*∗*^ *p* < 0.01 versus normal; *n* = 40 per group.

**Table 2:** CTRP9 protected diabetic hearts against IR injury.

	Control		IR		IR+0.3		IR+1		IR+3	
	30 min	120 min	30 min	120 min	30 min	120 min	30 min	120 min	30 min	120 min
HR (bpm)	262.1 ± 11.69^*∗*^	263.9 ± 8.71	248.6 ± 11.75	244.8 ± 4.98	266.2 ± 8.23	263.6 ± 13.85^##^	258.4 ± 9.33	273.1 ± 13.90	268.7 ± 12.00	275.7 ± 14.46
CF (mL/min)	11.16 ± 0.709	12.64 ± 1.018	11.00 ± 0.640	7.69 ± 0.287^##^	10.83 ± 0.793	11.27 ± 0.260^##*∗∗*^	10.89 ± 0.637	12.66 ± 0.551^*∗∗*$^	10.24 ± 0.852	12.32 ± 0.614^*∗∗*$^
LVDP (mmHg)	139.0 ± 6.81	135.2 ± 9.36	135.8 ± 4.44	88.4 ± 2.50^##^	140.4 ± 4.76	93.6 ± 6.29^##^	137.7 ± 6.88	108.3 ± 6.19^#*∗∗*$$^	142.7 ± 6.10	117.5 ± 7.11^#*∗∗*$$^
+d_*P*_/d_*t*_ (mmHg/sec)	2354 ± 98.7	2375 ± 68.3	2523 ± 95.4	1857 ± 85.2^##^	24118 ± 88.1	1968 ± 76.9^##^	2457 ± 116.1	2266 ± 106.7^#*∗∗*$$^	2547 ± 108.0	2307 ± 98.7^#*∗∗*$$^
−d_*P*_/d_*t*_ (mmHg/sec)	−2433 ± 85.9	−2369 ± 218.6	−2370 ± 97.1	−1735 ± 78.3^##^	−2398 ± 117.8	−1941 ± 109.1^##^	−2548 ± 122.5	−2261 ± 99.5^#*∗∗*$$^	−2608 ± 110.5	−2199 ± 117.5^#*∗∗*$$^
RPP	36418 ± 1014	35515 ± 1112	33487 ± 978	21477 ± 983^##^	37244 ± 1041	22459 ± 996^##^	35346 ± 1132	29484 ± 985^#*∗∗*$$^	38056 ± 1175	32177 ± 1107^#*∗∗*$$^

Isolated hearts from diabetic rats were subjected to IR or control protocol via a Langendorff apparatus at the presence of different dosage of globular CTRP9 (0.3, 1, and 3 *μ*g/mL for IR+0.3, IR+1, and IR+3 groups, resp.) or equal amount of KH buffer (control), and heart rate (HR), coronary flow (CF), left ventricular developing pressure (LVDP), maximum and minimum electronically differentiated derivative of left ventricular pressure (±d_*P*_ */*d_*t*_) and rate-pressure product (RPP) were compared at the end of reperfusion (120 min). ^#^ *p* < 0.05, ^##^ *p* < 0.01 versus control, ^*∗*^ *p* < 0.05, ^*∗∗*^ *p* < 0.01 versus IR, ^$^ *p* < 0.05, ^$$^ *p* < 0.01 versus IR+0.3, and *n* = 10 per group.
