# Supplementary figures and images for: C1q/TNF-Related Protein 9 Protects Diabetic Rat Heart against Ischemia Reperfusion Injury: Role of Endoplasmic Reticulum Stress
Source: Oxid Med Cell Longev. 2016 Oct 4;2016:1902025. doi: 10.1155/2016/1902025 (PMC5067328; doi:10.1155/2016/1902025)

## Supplementary Figure 1

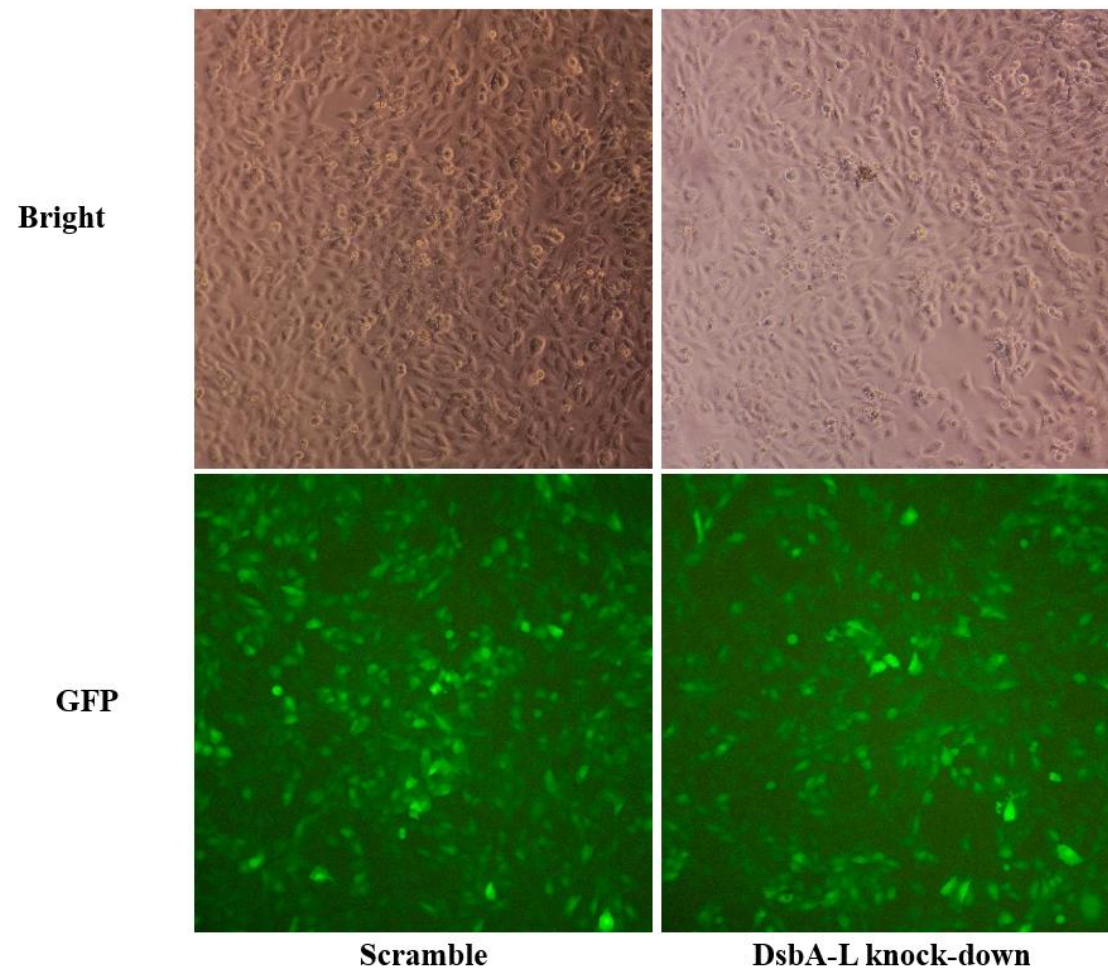

## Supplementary Figure 2

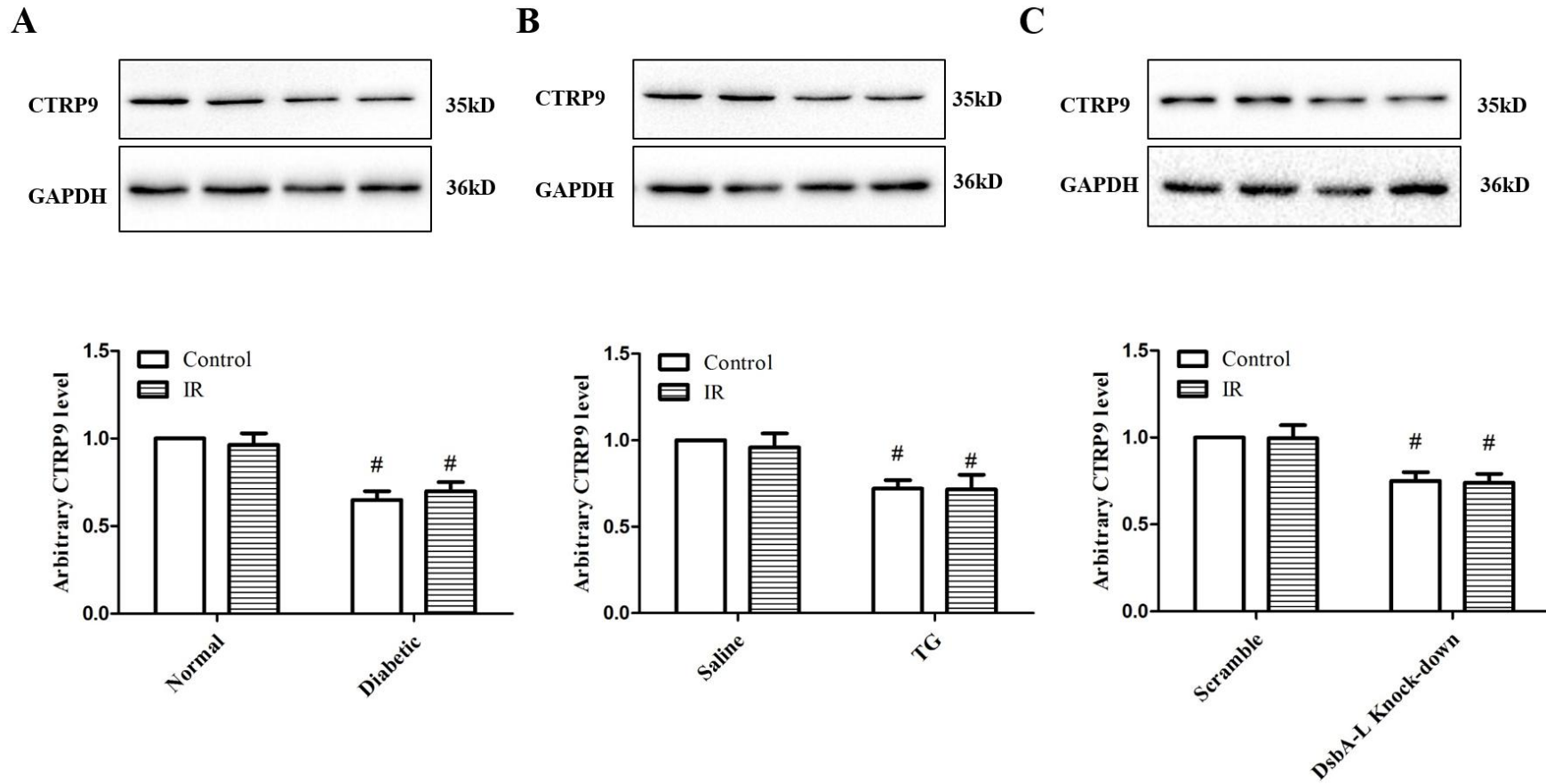

Supplement: Supplementary file 1 — Forty-eight hours after DsbA-L RNAi recombinant plasmids and non-specific scramble sequence were transfected in rat cardiomyocytes, transfection efficiency was shown as the expression of GFP in the recombinant plasmids (Supplementary Figure 1). Isolated hearts from normal or HFD induced type 2 diabetic rats were subjected to IR protocol and cardiac CTRP9 expression were examined at the end of reperfusion (Supplementary Figure 2A). TG or saline treated cardiomyocytes were subjected to SIR and cellular CTRP9 expression were examined at the end of reperfusion (Supplementary Figure 2B). Cardiomyocytes transfected with DsbA-L RNAi recombinant plasmids or non-specific scramble sequence were subjected to SIR and cellular CTRP9 expression were examined at the end of reperfusion (Supplementary Figure 2C). Representative images (upper panel) and bar graphs (lower panel) of cardiac CTRP9 determined by Western blots were shown. GAPDH was used as loading control. The results were expressed as the mean ± SEM, n = 8 per group, # p < 0.05. [file 1902025.f1.pdf]
